# Phytochemical Investigations, Antioxidant and Insecticidal Properties of Essential Oil and Extracts from the Aerial Parts of *Pelargonium graveolens* from Morocco

**DOI:** 10.3390/molecules29174036

**Published:** 2024-08-26

**Authors:** Zakya M’hamdi, Federica Davì, Mohammed Elhourri, Ali Amechrouq, Fabio Mondello, Francesco Cacciola, Roberto Laganà Vinci, Luigi Mondello, Natalizia Miceli, Maria Fernanda Taviano

**Affiliations:** 1 Laboratory of Molecular Chemistry and Natural Substances, Faculty of Science, Moulay Ismail University, B.P. 11201, Zitoune, Meknes 50050, Morocco; zakyamhamdi1997@gmail.com (Z.M.); med.elhourri@gmail.com (M.E.); a.amechrouq@umi.ac.ma (A.A.); 2 Department of Chemical, Biological, Pharmaceutical and Environmental Sciences, University of Messina, 98166 Messina, Italy; federica.davi@studenti.unime.it (F.D.); fabio.mondello@unime.it (F.M.); mtaviano@unime.it (M.F.T.); 3 Foundation “Prof. Antonio Imbesi”, University of Messina, 98122 Messina, Italy; 4 Department of Biomedical, Dental, Morphological and Functional Imaging Sciences, University of Messina, 98125 Messina, Italy; francesco.cacciola@unime.it; 5 C/o Messina Institute of Technology (MeIT), Department of Chemical, Biological, Pharmaceutical and Environmental Sciences, Former Veterinary School, University of Messina, 98168 Messina, Italylmondello@unime.it (L.M.); 6 Chromaleont s.r.l., C/o Messina Institute of Technology (MeIT), Department of Chemical, Biological, Pharmaceutical and Environmental Sciences, Former Veterinary School, University of Messina, 98168 Messina, Italy

**Keywords:** *Pelargonium graveolens* L’Hér., HPLC-PDA/ESI-MS, antioxidant activity, botanical insecticide, *Sitophilus oryzae* L., phenolic compounds

## Abstract

The essential oil and the aqueous and ethanolic extracts obtained from the aerial parts of *Pelargonium graveolens* cultivated in Morocco were studied for their antioxidant and insecticidal activity against rice weevils (*Sitophylus oryzae*). The total phenolic content of the extracts was determined by a spectrophotometric method and the phenolic compounds were extensively characterized by HPLC-PDA/ESI-MS. To evaluate antioxidant potential, three in vitro assays were used. In the DPPH test, the ethanolic extract was the most active, followed by the aqueous extract and the essential oil. In the reducing power assay, excellent activity was highlighted for both extracts, while in the Fe^2+^ chelating activity assay, weak activity was observed for both the essential oil and the ethanolic extract and no activity for the aqueous extract. Concerning insecticide activity, the toxicity of the essential oil and the extracts was tested against rice weevils; the lethal concentrations LC_50_ and LC_99_ were determined, as well as the lethal time required for the death of 50% (LT_50_) and 99% (LT_99_) of the weevils. The essential oil had the highest activity; 100% mortality of *S. oryzae* was observed around 5, 9, and 8 days for the essential oil and the aqueous and ethanolic extracts, respectively.

## 1. Introduction

The main problems affecting food during production, storage, and distribution are deterioration due to oxidation and attacks by pests. To protect foods from these effects, many synthetic chemicals are widely used, causing injury to non-target organisms as well as human and environmental health problems [1].

The use of plant-derived compounds instead of synthetic additives may be desirable, and there has been considerable interest in the isolation and development of new natural bioactive compounds. Phytochemicals are considered attractive due to their low cost, availability in large quantities from raw materials, biodegradability, and safety to human health and the environment [2]. In this direction, plant extracts and essential oils (EOs) stand out for their effectiveness throughout the world, while exploring the bioactivity of phytochemical compounds has proved to be an effective and more feasible means of controlling zoonotic diseases and reducing the microbial resistance index [3,4]. In recent years, EOs have effectively controlled stored product pests [5], as they contain monoterpenoid compounds that are toxic to insects by damaging their nervous systems [6].

The rice weevil (*Sitophilus oryzae* L.) is one of the most destructive pests of stored cereals and processed cereal products worldwide [7]. Indeed, several research studies have focused on the insecticidal and repellent activities of essential oils (EOs) and extracts from many plant species against rice weevils [8,9].

*Pelargonium graveolens* L’Hér. or “Geranium pink”, belonging to the Geraniaceae family, is a perennial aromatic shrub native to South Africa, Zimbabwe, and Mozambique, and widely cultivated in Russia, Egypt, Algeria, Morocco, Congo, Japan, Central America, and southern Europe (Spain, Italy, and France) [10]. This species is also used as a decoration and as a remedy in African, European, Chinese, Iranian, Indian, and Arabic traditional medicine [11,12]. It is well-known for its fragrance, and its EO, rich in geranial, (Z)-rose oxide, isomenthone, and linalool, is widely used as a pharmaceutical, cosmetic, and flavoring agent, as well as in folkloric foods and aromatherapy industries [13]. Geranium EO has historically been used to treat dysentery, hemorrhoids, inflammation, heavy menstrual flows, and even cancer. In French folk medicine, it is employed against diabetes, diarrhea, gallbladder problems, gastric ulcers, jaundice, liver problems, sterility, and urinary stones [14]. The pounded leaves are used to treat skin diseases (wounds and sores); the leaf decoction or infusion is employed against gastrointestinal disorders (constipation, intestinal cramps, and dysentery), hyperglycemia, and to relieve inflammatory and pain-associated ailments (i.e., headache and neuralgia), as well as those of the respiratory system (cold and cough). The decoction of the root is utilized against fever and tuberculosis; whereas the root infusion works against diarrhea and backache [15]. Several studies have confirmed that *P. graveolens* has a wide range of pharmacological effects, including anti-inflammatory and anticancer [16], anti-parasitic [17], anti-tuberculosis [18], and analgesic [19] effects. The plant has also been reported to have antimicrobial activity against many pathogenic bacteria and fungi [20,21]. Many chemical constituents such as volatile compounds, terpenoids, flavonoids, coumarins, phenolic acids, and tannins have been isolated from this species [12]. The research on *P. graveolens* is intensively focused on the chemical composition of the EO, mostly characterized by monoterpenes and sesquiterpenes (oxygenated and non-oxygenated). Oxygenated monoterpenes exist in a higher concentration than non-oxygenated monoterpenes, and the predominant ones are β-citronellol, geraniol, linalool, and isomenthone. Oxygenated sesquiterpenes are less abundant than non-oxygenated ones, including δ-selinene, β-caryophyllene, guaia-6,9-diene, and α-humulene [22,23,24].

Previously, some co-authors of this work characterized the chemical composition of the essential oil obtained from the aerial parts of *P. graveolens* grown in Er-Rachidia, Morocco. Using GC/MS analysis, epi-γ-eudesmol (16.67%), geraniol (12.54%), β-citronellol (12.34%), citronellyl formate (7.70%), geranyl tiglate (5.21%), and linalool (4.06%) were found to be the major compounds [25]. In continuation of the previous study, the present work was undertaken to investigate the antioxidant and insecticidal properties of the essential oil, as well as of the ethanolic and aqueous extracts from the aerial parts of this species. The antioxidant properties were examined by means of different in vitro systems: DPPH scavenging, reducing power, and ferrous ion (Fe^2+^)-chelating activity, and the insecticidal activity was evaluated against *S. oryzae*. In addition, the phenolic content of the ethanolic and aqueous extracts was determined by a Folin–Ciocalteu assay and characterized by HPLC-PDA/ESI-MS analysis.

## 2. Results and Discussion

### 2.1. Phytochemical Investigations

#### 2.1.1. Determination of Total Phenolic Content

Polyphenols are strong antioxidants widely distributed in nature in the form of secondary plant metabolites. They are classified into different subclasses based on the arrangement and the number of phenolic rings present, as well as the functional groups associated with these phenolic rings. Their antioxidant property is due to their ability to scavenge free radicals, donate hydrogen atoms or electrons, or chelate metal cations [26,27,28].

In the present work, the total phenolic content of the aqueous and ethanolic extracts of *P. graveolens* was estimated spectrophotometrically by the Folin–Ciocâlteu method, extensively used to quantify polyphenols in plant-derived extracts, as well as foods and drinks [29,30].

The results, reported in Table 1, show that the total phenolic content was found to be higher in the ethanolic extract, resulting in more than double that of the aqueous extract.

The total phenolic content of the extracts turned out to be higher than that previously reported for various extracts obtained from *P. graveolens*. Ćavar and Maksimović [23] found a much lower phenolic content in the aqueous extracts (hydrosols) obtained from leaves and stems of this species cultivated in Bosnia (34.88 ± 2.00 and 102.44 ± 1.63 mg GAE/g, respectively). A comparative study undertaken by Pradeepa et al. [31] on *P. graveolens* leaves collected in India showed that ethanolic extract, obtained by Soxhlet, had the highest total phenolic content (123.75 ± 8.25 mg GAE/g), followed by acetone (107.25 ± 4.25 mg GAE/g) and then methanolic (100.65 ± 4.90 mg GAE/g) and aqueous (24.75 ± 5.62 mg GAE/g) extracts. A similar work was conducted on extracts of leaves and flowers of *P. graveolens* from Tunisia using different solvents; the most abundant content was found in leaf and flower 80% methanol extracts (142.71 ± 3.83 mg GAE/g and 129.2 ± 2.60 mg GAE/g, respectively), followed by 80% ethanol extracts (136.54 ± 1.2 mg GAE/g and 118.05 ± 2.1 mg GAE/g, respectively) and water extracts (92.77 ± 2.50 and 55.44 ± 1.30 mgGAE7g, respectively) [32]. In another work conducted on aerial parts, aqueous extracts were obtained by the infusion and decoction of this species from Tunisia, and the phenolic content was found to be 27.05 ± 0.53 and 31.20 ± 0.58 mg GAE/g, respectively [33].

The extracts investigated in this study were obtained by using the Soxhlet extraction technique, which is known to offer numerous advantages such as high yields with a much lower volume of solvent. From comparisons with previous studies, it is evident that this technique (using ethanol as a solvent) represents an efficient system to recover a high content of phenolic compounds; notably, the extracts obtained from *P. graveolens* from Morocco are a richer source of phenolic compounds than those from the same species grown in other geographical areas.

#### 2.1.2. Identification of Phenolic Compounds by HPLC-PDA/ESI-MS

**Figure 1:**
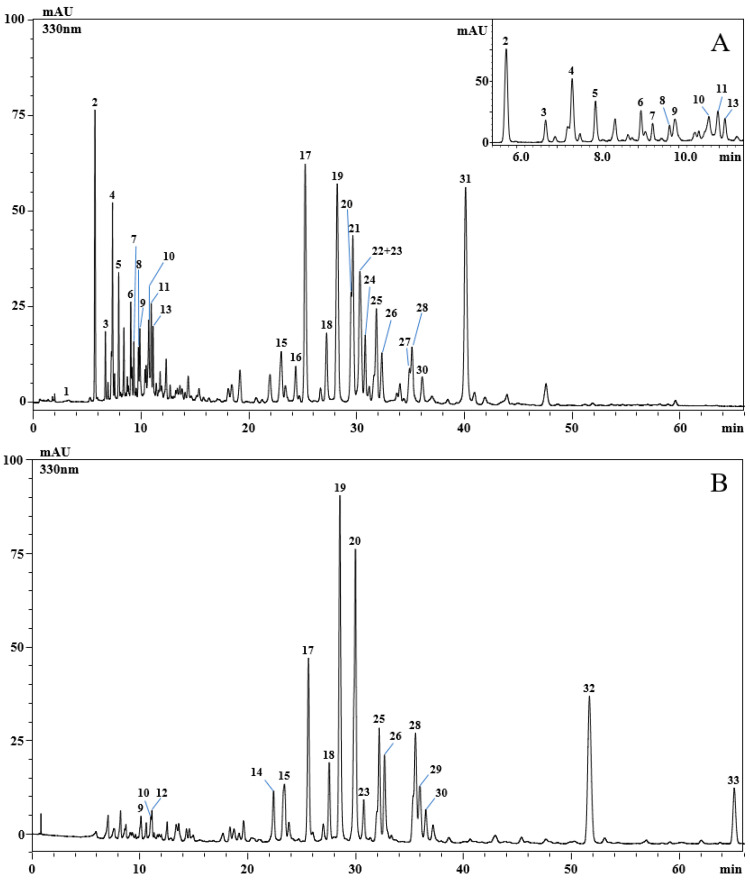
HPLC-PDA chromatograms of the phenolic compounds, extracted at 330 nm. Aqueous extract (**A**) and ethanolic extract (**B**) of *Pelargonium graveolens*. For peak identification, see Table 2.

Analysis of the phenolic profile of the aqueous and ethanolic extracts obtained from aerial parts of *P. graveolens* was carried out by using high-performance liquid chromatography coupled to a photodiode array and electrospray ionization mass spectrometry. A total of thirty-three phenolic compounds were detected (Figure 1A,B and Table 2).

In particular, most of them belonged to the flavonoid class, while only eight were phenolic acids. Of the flavonoids, eight were kaempferol derivatives, seven were quercetin derivatives, and four were myricetin derivatives. The eight phenolic acids were gallic acid, caffeoylglucaric acid, caftaric acid, feruloylglucaric acid, caffeoylquinic acid, caffeic acid, caffeoylhydroxycitric acid, and rosmarinic acid.

The results of the HPLC analysis of *P. graveolens* extracts have shown qualitative and quantitative differences in the phenolic content. Analysis of the ethanolic extract displayed 17 detected compounds. The main compounds were quercetin hexosyl-rhamnoside (9.09 ± 0.049 mg/g; peak 19), quercetin hexosyl-rhamnoside (8.63 ± 0.083 mg/g; peak 20), quercetin (5.45 ± 0.002 mg/g; peak 32), quercetin hexosyl-pentoside (4.41 ± 0.056 mg/g; peak 17), and quercetin 3-O-pentoside (3.09 ± 0.034 mg/g; peak 25). The remaining detected compounds were less than 2 mg/g, and two compounds were detected but not quantified. On the other hand, analysis of the aqueous extract of *P. graveolens* revealed 28 compounds, of which the major compounds were rosmarinic acid (8.59 ± 0.017 mg/g; peak 31), quercetin hexosyl-rhamnoside (4.44 ± 0.004 mg/g; peak 19), quercetin hexosyl-pentoside (4.36 ± 0.006 mg/g; peak 17), caffeoylglucaric acid (3.39 ± 0.011 mg/g; peak 2), kaempferol hexuronide and kaempferol hexosyl-pentoside (2.84 ± 0.010 mg/g; peak 22 and 23, respectively), quercetin hexoside (2.63 ± 0.034 mg/g; peak 21), and caffeoyl glucuronide (2.09 ± 0.044 mg/g; peak 4), while the other compounds were less than 2 mg/g.

Very few studies have investigated the phenolic composition of *P. graveolens* [34,35,36]; our results agree with those reported by Androutsopoulou [35] and Al-Sayed [36], who found quercetin and kaempferol derivatives to be the main phenolics detected in leaf extracts of *P. graveolens* from Greece and Egypt, respectively. Notably, this is the first work reporting an extensive characterization of the phenolic profile of aerial parts of this species growing in Morocco.

**Table 2:** Semi-quantification of phenolic compounds in aqueous and ethanolic extracts of the aerial parts of *Pelargonium graveolens* through LC-PDA/ESI-MS analysis. Quantification of phenolic compounds was reported in mg/g of dried extract ± SD (*n* = 3).

Peak N.	Compound	t_R_ (min)	UV max (nm)	[M − H]^−^	Aqueous Extract	Ethanolic Extract	Ref.
1	Gallic acid	2.91	270	169	0.60 ± 0.000	-	Std.
2	Caffeoylglucaric acid	5.74	326	371, 179	3.39 ± 0.011	-	[37]
3	Unknown	6.32	279	395, 197	X	-	-
4	Caffeoyl glucuronide	7.35	288, 312	355	2.09 ± 0.044	-	-
5	Caftaric acid	7.94	325	311, 179	1.13 ± 0.035	-	[38]
6	Feruloylglucaric acid	9.06	325	385, 193	0.72 ± 0.011	-	-
7	Sinapoylglucose	9.36	281, 322	385, 223	0.30 ± 0.010	-	-
8	Caffeoylglucose	9.77	323	341, 179	0.30 ± 0.003	-	-
9	Unknown	9.90	312	293	X	X	-
10	Caffeoylquinic acid	10.80	324	353, 191, 179	1.11 ± 0.002	0.23 ± 0.016	Std.
11	Caffeic acid	10.96	322	179	0.92 ± 0.012	-	Std.
12	Unknown	11.01	282	325	-	X	-
13	Caffeoylhydroxycitric acid	11.14	312	369	0.48 ± 0.003	-	-
14	Myricetin hexoside	22.40	260 sh, 354	479, 317	-	1.16 ± 0.000	[37]
15	Myricetin rhamnosyl-hexoside	23.42	262 sh, 353	625, 479, 317	1.06 ± 0.004	1.90 ± 0.022	[37]
16	Quercetin hexuronide	24.38	276, 343	477, 301	0.37 ± 0.011	-	[37]
17	Quercetin hexosyl-Pentoside	25.26	255, 353	595, 463, 301	4.36 ± 0.006	4.41 ± 0.056	[37]
18	Myricetin 3-O-rhamnoside	27.24	263, 348	463, 317	0.96 ± 0.006	1.49 ± 0.003	[35]
19	Quercetin hexosyl-rhamnoside	28.23	254, 353	609, 463, 301	4.44 ± 0.004	9.09 ± 0.049	[37]
20	Quercetin hexosyl-rhamnoside	29.53	256, 352	609, 463, 301	1.34 ± 0.041	8.63 ± 0.083	[37]
21	Quercetin hexoside	29.68	254, 352	463, 301	2.63 ± 0.034	-	[37]
22	Kaempferol hexuronide	30.34	261, 347	461, 285	2.84 ± 0.010	-	-
23	Kaempferol hexosyl-pentoside	30.79	265, 345	579, 447, 285		0.65 ± 0.008	[39]
24	Kaempferol hexosyl-rhamnoside	30.82	266, 347	593, 447, 285	0.81 ± 0.000	-	[37]
25	Quercetin 3-O-pentoside	31.86	255, 353	433, 301	1.71 ± 0.008	3.09 ± 0.034	[35]
26	Kaempferol 3-O-glucoside	32.36	264, 344	447, 285	0.70 ± 0.001	1.71 ± 0.016	Std.
7	Kaempferol hexosyl-rhamnoside	34.93	265, 343	593, 447, 285	0.37 ± 0.017	-	[37]
28	Kaempferol galactoside	35.16	264, 344	447, 285	0.92 ± 0.015	3.29 ± 0.033	[37]
29	Myricetin	35.99	252 sh, 370	317	-	1.38 ± 0.017	Std.
30	Kaempferol 3-O-pentoside	36.53	265, 345	417, 285	0.34 ± 0.003	0.70 ± 0.009	[35]
31	Rosmarinic acid	40.13	328	359, 161	8.59 ± 0.017	-	[40]
32	Quercetin	51.69	254, 369	301	-	5.45 ± 0.002	Std.
33	Kaempferol	65.07	265, 366	285	-	1.48 ± 0.007	Std.

X: detected but not quantified; sh: wavelength shoulder.

### 2.2. Antioxidant Activity

The antioxidant properties of the aqueous and ethanolic extracts and EO of *P. graveolens* were established using three in vitro tests to evaluate the different mechanisms through which the diverse antioxidant compounds contained in the phytocomplexes could exert their effect. The primary antioxidant properties were evaluated by a DPPH assay, based on hydrogen atom transfer (HAT) and single-electron transfer (SET) mechanisms and reducing power, and a SET-based assay; the ferrous ion (Fe^2+^)-chelating activity assay was utilized to determine the secondary antioxidant properties.

**Figure 2:**
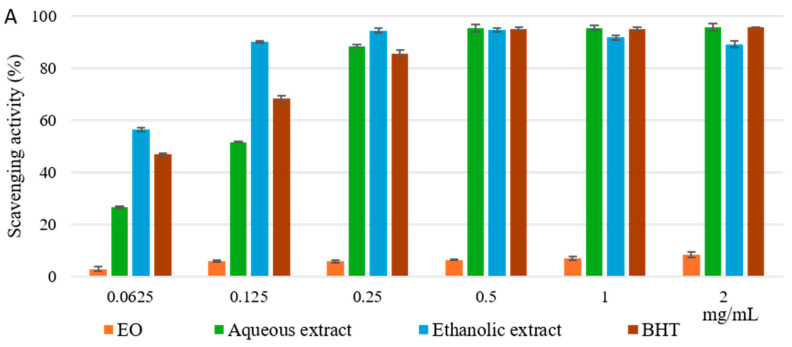

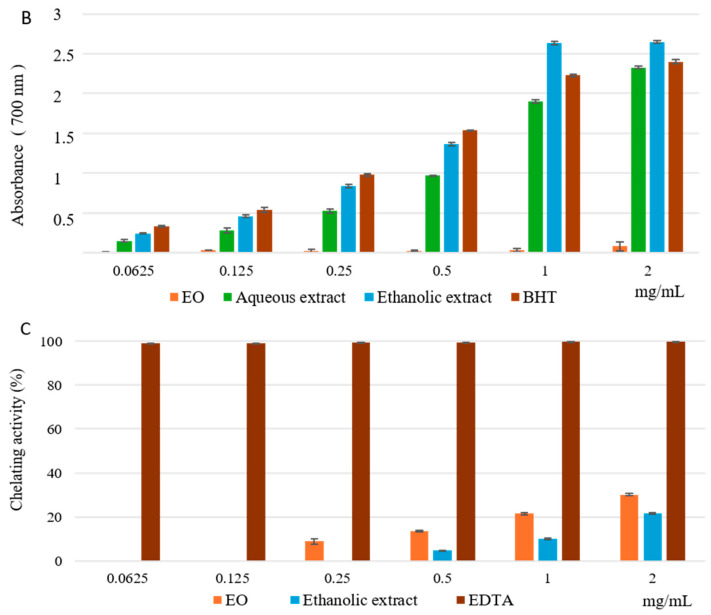
Free radical scavenging activity (DPPH assay) (**A**), reducing power (**B**), and ferrous ion-chelating activity (**C**) of EO and ethanolic and aqueous extracts obtained from aerial parts of *Pelargonium graveolens*. Values are expressed as the mean ± SD (n = 3).

The results of the DPPH test, utilized to determine the scavenging properties of free radicals, are shown in Figure 2A. Both aqueous and ethanolic extracts exhibited excellent radical scavenging activity; the ethanolic extract at the lowest concentrations (0.0625 to 0.250 mg/mL) showed a higher effect than the reference standard BHT, reaching its maximum activity, above 90%, at the concentration of 0.250 mg/mL. On the other hand, the EO showed very low activity. This is also confirmed by the calculated IC_50_ values equal to 0.05 ± 0.011 mg/mL for ethanolic extract, which is better than BHT (IC_50_ = 0.07 ± 0.01 mg/mL), followed by the aqueous extract (IC_50_ = 0.13 ± 0.01 mg/mL) and EO (IC_50_ > 2 mg/mL) (Table 1). Figure 2B shows the results of the reducing power assay. Excellent reducing capabilities were highlighted for the ethanolic and aqueous extracts compared to the reference standard BHT. Ethanolic extract from the 1 mg/mL concentration was more active than the standard. However, no statistically significant difference between the ASE/mL values of aqueous and ethanolic extracts (3.01 ± 0.03 and 1.92 ± 0.04 ASE/mL, respectively) compared to the BHT (1.44 ± 0.02 ASE/mL) was found, as shown in Table 1. Instead, the EO showed weak reducing power (21.77 ± 2.17 ASE/mL). In the Fe^2+^ chelating activity assay, the EO and the ethanolic extract showed low activity compared to the reference standard EDTA (Figure 2C), also demonstrated by IC_50_ values > 2 mg/mL for both (Table 1). On the contrary, the aqueous extract showed no activity.

The results of the antioxidant tests indicate that the aqueous and ethanolic extracts showed excellent primary antioxidant properties; on the contrary, the EO has shown weak antioxidant properties, both primary and secondary. The primary antioxidant properties could be mainly attributed to the phenolic compounds detected in the extracts by HPLC-PDA/ESI-MS analysis. Flavonoids and phenolic acids, the largest classes of plant phenolics, are effective antioxidants; the antioxidant activity of these compounds is mainly due to their redox properties and chemical structure, which contribute to their ability to inhibit lipoxygenase and scavenging free radicals [41,42,43]. The best radical scavenging activity of the ethanolic extract could be related to the presence of the flavonols quercetin and myricetin and their derivatives, whose antioxidant properties have been widely demonstrated [44,45,46]. These compounds were found in larger quantities in the ethanolic extract than the aqueous one.

Several previous works indicated *P. graveolens* as a potential source of antioxidant compounds. Referring to the literature, studies on the antioxidant activity of this species were conducted mainly on the essential oil, showing a strong antioxidant effect, which does not agree with our results [10,32,47,48,49]. On the contrary, our findings are similar to those reported by Ćavar et al. [23], showing very weak reactivity in the scavenging of DPPH radicals in the essential oils from the air-dried leaves and stems of *P. graveolens*.

Furthermore, Dimitrova et al. [50] and Ennaifer et al. [33,51] reported the remarkable antioxidant capacity of aqueous extracts of this species. El Aanachi et al. [13] showed the activities of extracts from aerial parts (n-hexane, dichloromethane, and methanol) of *P. graveolens* by various antioxidant assays, including DPPH scavenging, reducing power, and iron chelation. Strong antioxidant activity was demonstrated by the extracts, particularly the methanol extract, which was the most powerful.

### 2.3. Insecticidal Activity on Adult Sitophilus oryzae

**Figure 3:**
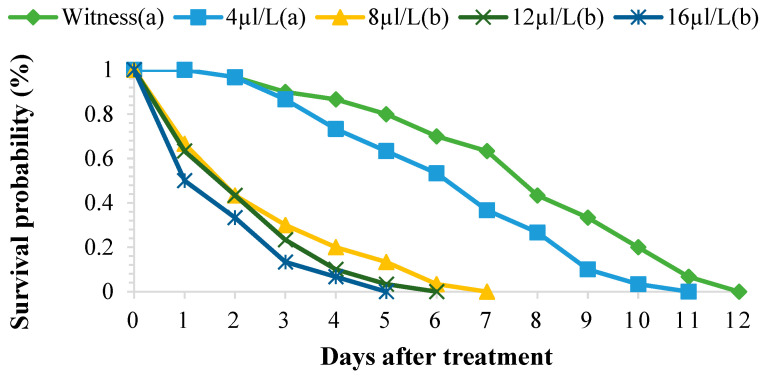
Survival of adult *Sitophilus oryzae* treated with the EO of *Pelargonium graveolens*. Survivors with the same lower-case letter did not differ statistically from one another (Scheffé test, *p* ≤ 0.05), while the others were different.

The EO of *P. graveolens* at different concentrations (4, 8, 12, and 16 µL/L of air) significantly affected the survival of *S. oryzae* adults. In the treated batches, this survival ranged between 1 and 10 days for the concentration of 16 µL/L of air, whereas in the control batch, this parameter varied between 3 and 12 days. The toxicity of EO depends on the concentration and duration of exposure (Figure 3). The survival times of 50% of the adults exposed to different concentrations of EO varied from one day to around five days, whereas in the control batch, the adults lived for an average of 12 days. The TL_50_ and TL_99_ were negatively correlated with the concentrations of EO tested (Table 3). The toxicological parameters of the EO tested are shown in Table 4. After three days of treatment, the LC_50_ and LC_99_ concentration values were 19.22 µL/L and 76.42 µL/L, respectively.

Abd El-Salam [52] found that the EOs of *Cymbopogon flexuosus* and *Melaleuca alternifolia* had potent toxicity against *S. oryzae*. The LC_50_ of these essential oils were, respectively, 31.0, 36.0, and 69.6 µL/L after three days of treatment, while the LC_50_ of the *P. graveolens* EO studied was 19.22 µL/L, showing that *S. oryzae* was more sensitive to this oil. In addition, Mesbah et al. [53] evaluated the *S. oryzae* contact toxicity of the EO from *P. graveolens* and prepared nanoemulsions. The results showed that the nanoemulsion had the best activity (LC_50_ = 2.29 ppm/cm^2^) against adult *S. oryzae* after 72 h, whereas the EO was found to be less toxic, (LC_50_ = 67.662 ppm/cm^2^). A study carried out by Jayakumar et al. [54] assayed the fumigant and the repellent effect of geranium EO on *S. oryzae* and found a fumigant effect on rice weevils. Seada et al. [55] evaluated the contact toxicity of *P. graveolens* and found that geranium oil had the highest repellent activity against *S. oryzae*, followed by fennel and basil oils. The results of the study carried out by Arab et al. [56] indicated that geranium stripping oil was highly toxic against adult *S. oryzae*. after 24 h of exposure (LC_50_ = 1310.4 mg/L), in agreement with our findings.

**Figure 4:**
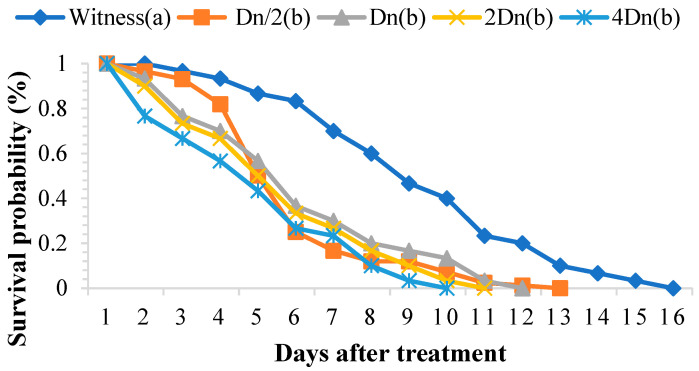
Survival of adult *Sitophilus oryzae* treated with the ethanolic extract of *Pelargonium graveolens.* Survivors with the same lower-case letter did not differ statistically from one another (Scheffé test *p* ≤ 0.05), while the others were different.

The ethanolic extract of *P. graveolens* significantly affected the survival of adult *S. oryzae*. In the treated batches, weevil survival ranged from one to eleven days, whereas in the control batch, this parameter fluctuated between two and fifteen days. The toxicity of the ethanolic extract depended on the concentration and duration of exposure (Figure 4). The TL_50_ and TL_99_ were negatively correlated with the concentrations tested (Table 5).

The toxicity parameters of the ethanolic extract of *P. graveolens* are summarized in Table 6. The calculated lethal concentrations LC_50_ and LC_99_ reveal that adults of *S. oryzae* are very sensitive to this extract. The extreme values of LC_50_ and LC_99_ vary according to the duration of exposure (Table 6).

**Figure 5:**
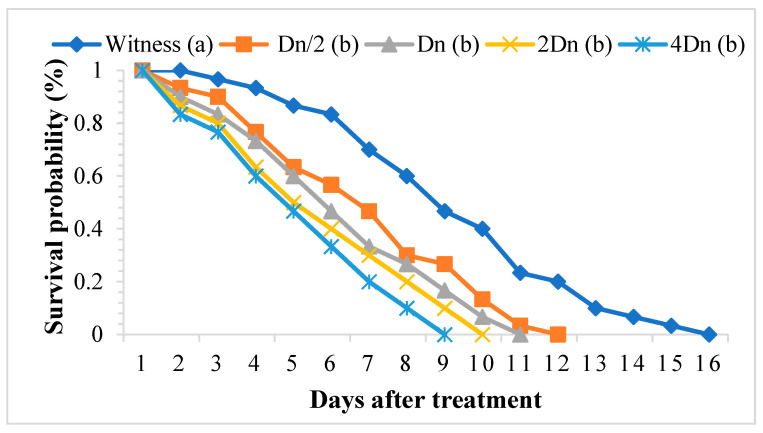
Survival of adult *Sitophilus oryzae* treated with the aqueous extract of *Pelargonium graveolens.* Survivors with the same lower-case letter did not differ statistically from one another (Scheffé test *p* ≤ 0.05), while the others were different.

The aqueous extract of *P. graveolens* significantly affected the survival of adult *S. oryzae*. In the treated batches, weevil survival ranged from one to eleven days, whereas in the control batch, this parameter fluctuated between two and fifteen days. The toxicity of the aqueous extract of *P. graveolens* depended on the concentration and duration of exposure (Figure 5). The TL_50_ and TL_99_ were negatively correlated with the concentrations tested (Table 7).

The toxicity parameters of the aqueous extract of *P. graveolens* are summarized in Table 8. The calculated lethal concentrations LC_50_ and LC_99_ reveal that adults of *S. oryzae* are more sensitive to this aqueous extract. The extreme values of LC_50_ and LC_99_ vary according to the duration of exposure.

Overall, the obtained results highlighted the strongest toxicity against *S. oryzae* for *P. graveolens* EO. The strong insecticidal action of EO could depend on the presence of some components contained in high amounts such as monoterpenoids [25]. These compounds are severely poisonous to insects and have repellent and antifeedant qualities; for this reason, they have been explored as possible pest control agents [57]. In particular, this effect could depend mainly on geraniol, citronellol, and linalool detected in great concentrations in the EO and whose toxicity against rice weevils has been demonstrated [56]. The findings of the present study indicate that this EO can provide an alternative source of insect control agents because it contains a range of bioactive chemicals, most of which are selective and have little or no harmful effect on the environment and non-target organisms including humans. EO-based formulations can be used as alternative tools in stored grain insect management [58].

Interestingly, even the ethanolic and the aqueous extracts, rich in phenolics, exhibited toxicity against rice weevils, with the former being more active than the latter. The effects of plant extracts and their active constituents, including flavonoids and phenolic acids, against stored product insect pests have been previously reported; indeed, several phenolic compounds were found to possess insecticidal activity against *S. oryzae* [59,60]. As far as we know, there are no data in the previous literature on the insecticidal activity of *P. graveolens* extracts against *S. oryzae*.

## 3. Materials and Methods

### 3.1. Plant Material and Extraction Procedure

The aerial parts of *P. graveolens* were harvested in May 2020 in the ksar Tizgaghine, 20 km from Tinjdad, in the region of Er-Rachidia, Morocco (31°55′55″ N, 4°25′28″ W). The plant was identified and confirmed by Professor Benkhnigue Ouafae at the Botanics and Plant Ecology Department of the Scientific Institute of Rabat, Morocco. The plant was deposited in the herbarium under the voucher number RAB 114766. The plant material was dried in a dry ventilated place for one month, then ground with an electric mill and kept in the shade in closed premises. A total of 30 g of powdered plant material was put in a cartridge and extracted with 250 mL of extraction solvent (ethanol or water) using a Soxhlet extractor for 6 h. Then, the solvent was evaporated using a rotary evaporator. The extraction yield of ethanolic and aqueous extracts was 18.26 and 22.25%, respectively.

The essential oil was extracted by hydro-distillation; 100 g of dry plant material was placed in 1.5 L of distilled water heated to 100 °C in a Clevenger-type apparatus. Distillation was performed for three hours after the first drop of distillate had been collected. The essential oil was dried with anhydrous sodium sulfate and stored at +4 °C in the dark. The extraction yield of the essential oil was 0.21%.

### 3.2. Phytochemical Investigations

#### 3.2.1. Determination of Total Phenolic Content

The total phenolic content of the aqueous and ethanolic extracts was determined by the Folin–Ciocâlteu colorimetric method as previously reported [61]. The results were obtained from the average of three independent determinations and expressed as mg gallic acid equivalent (GAE)/g extract (dw) ± standard deviation (SD).

#### 3.2.2. Phenolic Compounds Analysis by HPLC-PDA/ESI-MS

Analysis of phenolic compounds of the aqueous and ethanolic extracts was performed using high-performance liquid chromatography coupled with a photodiode array detector and electrospray ionization mass spectrometry (HPLC-PDA/ESI-MS) (Shimadzu, Kyoto, Japan). Chromatographic separation was carried out on an Ascentis Express C18 column (150 × 2.1 mm, 2.7 μm; Merck Life Science, Merck KGaA, Darmstadt, Germany) using, as the mobile phase, 0.1 % (*v*/*v*) acid formic in water (mobile phase A) and 0.1 % (*v*/*v*) acid formic in acetonitrile (mobile phase B). The gradient elution applied was: 0 min (0 % B), 10 min (10 % B), 20 min (11 % B), 30 min (15 % B), 50 min (18 % B), 65 min (23 % B), 70 min (100 % B), and 75 min (100 % B) at a flow rate of 0.5 mL/min. The column temperature was 30 °C and the injection volume was 2 μL. UV detection wavelengths were in the range of λ =190–400 nm. Positive and negative ion mass spectra were set as follows: scan range: *m*/*z* 100–800, nebulizing gas (N_2_) flow rate: 0.5 mL/min, drying gas (N_2_) flow rate: 15 L/min, interface temperature: 350 °C. LabSolutions software ver. 5.92 (Shimadzu, Kyoto, Japan) was used to control the LC-PDA-ESI-MS system and for data processing. The identification of phenolic compounds was made by comparison of retention times and UV–visible and mass spectra, and with co-standard injection data and data from the literature when available.

### 3.3. Antioxidant Activity

#### 3.3.1. DPPH Test

The 2,2-diphenyl-1-picrylhydrazyl (DPPH) test was used to determine the free radical scavenging activity of *P. graveolens* extracts and EO, according to the method of Ohnishi et al. [62], using butylated hydroxytoluene (BHT) as the reference standard. The results were obtained from the average of three independent experiments, and are reported as mean radical scavenging activity (%) ± SD and mean 50% inhibitory concentration (IC_50_) ± SD.

#### 3.3.2. Reducing Power Assay

The reducing power of *P. graveolens* extracts and EO was determined using the Fe^3+^-Fe^2+^ transformation method, according to the protocol of Oyaizu [63], using Ascorbic acid and BHT as reference standards. The results were obtained from the average of three independent experiments, and are expressed as mean absorbance values ± SD and ascorbic acid equivalent/mL (ASE/mL) ± SD.

#### 3.3.3. Ferrous Ions (Fe^2+^) Chelating Activity Assay

The chelating activity of *P. graveolens* extracts and EO was measured by evaluating their ability to inhibit the formation of the Fe^2+^-ferrozine complex, according to the method previously reported by Kumar et al. [64]. The results, obtained from the average of three independent experiments, are reported as the mean inhibition of ferrozine–(Fe^2+^) complex formation (%) ± SD and IC_50_ ± SD.

### 3.4. Insecticidal Activity

#### 3.4.1. *Sitophilus oryzae* Strain

The insects were derived from a strain isolated from wheat grains infested with *S. oryzae*. The grains were collected from a farmer in the Meknes region. The strain was grown in the laboratory in a ventilated room at 25–28 °C and 70% humidity. Mass rearing was carried out in glass jars with mesh lids, filled with durum wheat grains, to which a sufficient number of *S. oryzae* insects of undetermined sex were added. The pots were then left at room temperature. After one or two weeks of infestation, the adults were removed from the grains.

#### 3.4.2. Effect of the Essential Oil on Adult *Sitophilus oryzae*

*Pelargonium graveolens* EO oil fumigant was used in 2.5 L hermetically sealed transparent plastic boxes as an exposure chamber to test the essential oil’s toxicity against adult *S. oryzae*, using a modified version of the techniques outlined by El Idrissi et al. (2014) [65]. Five Petri dishes are placed in each box (ensuring five repetitions). Each repetition consists of ten *S. oryzae* adults. Five Petri dishes were placed, each replicate consisting of ten *S. oryzae* adults. The tests were carried out under rearing conditions. The EO was spread on Whatman-type filter paper, which was placed inside the exposure chamber. Four doses were applied: 4 µL, 8 µL, 12 µL, and 16 µL, and an untreated batch was used as a control. Mortality was monitored by counting dead insects from the first day of treatment until death.

#### 3.4.3. Effect of Ethanolic and Aqueous Extracts on Adult *Sitophilus oryzae*

The method outlined by Riffi et al. (2019) [66] was used to assess the fumigant effect of *P. graveolens* extracts against adult *S. oryzae*. Ten wheat burrows were introduced into Petri dishes containing 50 durum wheat seeds mixed with the ethanolic and aqueous extracts of the aerial part of *P. graveolens* at five selected doses (0; Dn/2; Dn; 2Dn; and 4Dn), either an extract weight of 0 g, 0.0078 g, 0.0156 g, 0.0321 g, and 0.0624 g, respectively, for the ethanolic extract or an extract weight of 0 g, 0.0127 g, 0.0254 g, 0.0508 g, and 0.1017 g, respectively, for the aqueous extract. The tests were carried out under the conditions of breeding for *S. oryzae*. Mortality control was done by enumerating dead insects from the first day of treatment to the death of all individuals. For each dose, the experiments were repeated three times.

#### 3.4.4. Data Analysis

The LC_50_ and LC_99_ were determined using the Finney probit method [67]. Mortalities were corrected using Abbott’s formula [68]. The lethal times required for the death of 50% (TL_50_) and 99% (TL_99_) of adults exposed to different concentrations of the essential oil and extracts were estimated.

### 3.5. Statistical Analysis

Statistical analysis of data regarding the antioxidant activity was carried out by using one-way analysis of variance (ANOVA) followed by the Tukey–Kramer multiple comparisons tests; conversely, the *t*-test was employed for total phenolic content data handling (GraphPad Prism Software for Science or Statistica 13.3 (TIBCO Software Co., Palo Alto, CA, USA)). In all the selected tests, *p*-values lower than 0.0001 were considered statistically significant. To compare the effects of the essential oil and the extracts tested on insecticidal activity, analysis of variance (ANOVA) followed by the 5% Scheffé test was performed using Excel version 2010 software.

## 4. Conclusions

In this contribution, the essential oil and the extracts (aqueous and ethanolic) obtained from the aerial parts of *Pelargonium graveolens* grown in Er-Rachidia, Morocco, have been assayed for their in vitro antioxidant activity and insecticidal properties against the rice weevil (*Sitophilus oryzae*), one of the most destructive pests of stored cereals and processed cereal products worldwide.

The results of the antioxidant tests showed the best activity for the ethanolic extract, followed by the aqueous one, whereas EO exhibited weak antioxidant properties, indicating that phenolic compounds play a major role in the observed effects. A thorough characterization of the phenolic profile of the aqueous and ethanolic extracts has been performed, which revealed quite a complex and different pattern, including phenolic acids and flavonoids. Differently, the essential oil displayed the strongest toxicity against *S. oryzae*, which could depend mainly on the presence of some monoterpenoids in high amounts. Notably, even the ethanolic and the aqueous extracts exhibited toxicity against rice weevils, with the former being more active than the latter, which could be related to phenolic compounds.

Based on the remarkable results achieved for antioxidant and insecticidal activity, the aerial parts of *P. graveolens* could be considered as an alternative source of bioactive compounds to be advantageously employed as botanical insecticides against several stored and processed product insect pests.

## Figures and Tables

**Table 1:** Quantitative determination of total phenolic content (TPC), free radical scavenging activity (DPPH assay), reducing power, and ferrous ion-chelating activity of essential oil and ethanolic and aqueous extracts obtained from the aerial parts of *Pelargonium graveolens*.

*Pelargonium graveolens*	TPC (mg GAE/g Extract)	DPPH IC_50_ (mg/mL)	Reducing Power ASE/mL	Chelating Activity Fe^2+^ IC_50_ (mg/mL)
EO	ND	>2 ^a^	21.77 ± 2.17 ^a^	>2 ^a^
Aqueous extract	156.42 ± 0.73 ^a^	0.13 ± 0.01 ^b^	3.01 ± 0.03 ^b^	NA
Ethanolic extract	385.09 ± 2.09 ^b^	0.05 ± 0.01 ^c^	1.92 ± 0.04 ^b^	>2 ^a^
Standard	-	BHT 0.07 ± 0.01 ^d^	BHT 1.44 ± 0.02 ^b^	EDTA 0.007 ± 0.001 ^b^

Values are expressed as the mean ± SD (n = 3). ND: Not determined. NA: Not active. ^a–d^ Different letters within the same column indicate significant differences between mean values (*p* < 0.0001).

**Table 3:** TL_50_ and TL_99_ of *Sitophilus oryzae* adults exposed to *Pelargonium graveolens* essential oil.

Concentrations (µL/L)	TL_50_	r > r (0.05; 2)	TL_99_	r > r (0.05; 2)
0	6.89		13.65	
4	5.58	−0.89	11.05	−0.89
8	3.18		6.30	
12	2.71		5.36	

**Table 4:** Toxicity parameters of essential oil of *Pelargonium graveolens* on *Sitophilus oryzae*.

Days after Treatment	Slope ± SE ^(1)^	χ^2^ Calculated <χ^2^ (0.05; 2) = 5.991	LC_50_ (µL/L) ^(2)^ [Confidence Interval]	LC_99_ (µL/L) ^(2)^ [Confidence Interval]
1	3.03 ± 0.71	4.36	36.78 [30.03; 53.74]	215.88 [109.33; 1312.13]
3	3.88 ± 0.81	3.70	19.22 [14.23; 23.25]	76.42 [53.353; 171.002]
4	4.11 ± 0.82	1.96	15.35 [10.96; 18.82]	56.40 [41.22; 108.74]
5	5.66 ± 1.22	0.30	13.79 [9.82; 16.78]	35.55 [27.78; 59.75]
6	8.02 ± 2.14	0.02	12.30 [8.73; 15.07]	23.99 [18.89; 43.78]

^(1)^ SE: Standard Error; ^(2)^ LC_50_ and LC_99_: Lethal concentrations, respectively, for 50% and 99% of the individuals used.

**Table 5:** TL_50_ and TL_99_ of *Sitophilus oryzae* adults exposed to *Pelargonium graveolens* ethanolic extract.

Concentrations (g/50 Seeds)	TL_50_	r > r (0.05; 2)	TL_99_	r > r (0.05; 2)
0	7.71		13.65	
Dn/2	5.53	−0.99	11.05	−0.99
Dn	5.05		6.30	
2 Dn	4.62		5.36	
4 Dn	4.15		8.21	

**Table 6:** Toxicity parameters of ethanolic extract of *Pelargonium graveolens* on *Sitophilus oryzae*.

Days after Treatment	Slope ±SE ^(1)^	χ^2^ Calculated <χ^2^ (0.05; 2) = 5.991	LC_50_ (g/50 Seeds) ^(2)^ [Confidence Interval]	LC_99_ (g/50 Seeds) ^(2)^ [Confidence Interval]
1	1.56 ± 0.71	0.33	10.63 [5.09; 922656.81]	329.52 [109.33; 1312.13]
6	1.54 ± 0.77	0.23	1.93 [0.00; 4.12]	62.13 [53.35; 171.002]
8	3.17 ± 1.20	2.01	1.38 [0.20; 2.13]	7.507 [41.22; 108.74]
9	3.47 ± 1.44	1.31	0.82 [0.03; 1.33]	3.86 [27.78; 59.75]

^(1)^ SE: Standard Error; ^(2)^ LC_50_ and LC_99_: Lethal concentrations, respectively, for 50% and 99% of the individuals used.

**Table 7:** TL_50_ and TL_99_ of *Sitophilus oryzae* adults exposed to *Pelargonium graveolens* aqueous extract.

Concentrations (g/50 Seeds)	TL_50_	r > r (0.05; 2)	TL_99_	r > r (0.05; 2)
0	7.53		14.90	
Dn/2	5.24	−0.99	10.38	−0.99
Dn	4.90		9.96	
2 Dn	4.47		8.85	
4 Dn	4.24		8.39	

**Table 8:** Toxicity parameters of the aqueous extract of *Pelargonium graveolens* on *Sitophilus oryzae*.

Days after Treatment	Slope ±SE ^(1)^	χ^2^ Calculated <χ^2^ (0.05; 2) = 5.991	LC_50_ (g/50 Seeds) ^(2)^ [Confidence Interval]	LC_99_ (g/50 Seeds) ^(2)^ [Confidence Interval]
1	1.89 ± 0.86	0.80	11.34 [5.09; 922,656.81]	192.63 [109.33; 1312.13]
2	1.83 ± 0.83	0.68	6.96 [0.00; 4.12]	129.83 [53.35; 171.002]
3	1.50 ± 0.76	0.18	6.30 [0.20; 2.13]	221.01 [41.22; 108.74]
8	2.20 ± 1.01	0.64	1.16 [0.03; 1.33]	13.21 [27.78; 59.75]

^(1)^ SE: Standard Error; ^(2)^ LC_50_ and LC_99_: Lethal concentrations, respectively, for 50% and 99% of the individuals used.

## Data Availability

The data presented in this study are available on request from the authors.
